# Tensile Properties of In Situ 3D Printed Glass Fiber-Reinforced PLA

**DOI:** 10.3390/polym15163436

**Published:** 2023-08-17

**Authors:** Khairul Izwan Ismail, Rayson Pang, Rehan Ahmed, Tze Chuen Yap

**Affiliations:** 1 School of Engineering and Physical Sciences, Heriot-Watt University Malaysia, No. 1, Jalan Venna P5/2, Precinct 5, Putrajaya 62200, Malaysia; r.pang@hw.ac.uk; 2 School of Engineering and Physical Sciences, Heriot-Watt University, Edinburgh EH14 4AS, UK; r.ahmed@hw.ac.uk

**Keywords:** 3D printing, fused deposition modelling, mechanical characterization, PLA composites, scanning electron microscopy, fiber-reinforced composite

## Abstract

A 3D printed composite via the fused filament fabrication (FFF) technique has potential to enhance the mechanical properties of FFF 3D printed parts. The most commonly employed techniques for 3D composite printing (method 1) utilized premixed composite filaments, where the fibers were integrated into thermoplastic materials prior to printing. In the second method (method 2), short fibers and thermoplastic were mixed together within the extruder of a 3D printer to form a composite part. However, no research has been conducted on method 3, which involves embedding short fibers into the printed object during the actual printing process. A novel approach concerning 3D printing in situ fiber-reinforced polymer (FRP) by embedding glass fibers between deposited layers during printing was proposed recently. An experimental investigation has been undertaken to evaluate the tensile behavior of the composites manufactured by the new manufacturing method. Neat polylactic acid (PLA) and three different glass fiber-reinforced polylactic acid (GFPLA) composites with 1.02%, 2.39%, and 4.98% glass fiber contents, respectively, were 3Dprinted. Tensile tests were conducted with five repetitions for each sample. The fracture surfaces of the samples were then observed under scanning electron microscopy (SEM). In addition, the porosities of the 3D printed samples were measured with a image processing software (ImageJ 1.53t). The result shows that the tensile strengths of GFPLA were higher than the neat PLA. The tensile strength of the composites increased from GFPLA-1 (with a 1.02% glass fiber content) to GFPLA-2.4 (with a 2.39% glass fiber content), but drastically dropped at GFPLA-5 (with a 4.98% glass fiber content). However, the tensile strength of GFPLA-5 is still higher than the neat PLA. The fracture surfaces of tensile samples were observed under scanning electron microscopy (SEM). The SEM images showed the average line width of the deposited material increased as glass fiber content increased, while layer height was maintained. The intralayer bond of the deposited filaments improved via the new fiber embedding method. Hence, the porosity area is reduced as glass fiber content increased.

## 1. Introduction

FFF, also known as fused deposition modeling (FDM), was invented and developed by Stratasys Inc. in the early 1990s and is the most widely used technique among all additive manufacturing (AM) technologies, showing high potential for fabricating plastic parts with the capacity to compete with conventional processing techniques [1]. The range of applications of FFF is extensive, ranging from medical treatment [2,3] and mold design [4], to automotive [5] and aerospace [6]. In the FFF process, the thermoplastic filament as feedstock is fed into a heating chamber via a stepping motor and extruded through the heated nozzle in a prescribed manner on a layer-by-layer basis [7,8]. Each deposited layer forms interlamellar bonds between the adjacent layers, which is then divided into two types: intralayer (within the same layer) and interlayer (between different layers).

Although the deposited filaments can be integrated into the adjacent-deposited filaments due to gravity and the force of the stepping motor, there still exist significant voids between the deposited filaments, which impairs the mechanical properties of the fabricated parts to a great degree, caused by the weak inter- and intralayer bonds between the deposited filaments [9]. On the other hand, the extruded filament cools quickly from the melting temperature, resulting in residual stress and weakening bonds between the two deposited filaments. Thermoplastics, such as PLA, acrylonitrile butadiene styrene (ABS), poly-ether-ether-ketone (PEEK), and polypropylene (PP) in the form of filament, are common polymers that have been consistently used with FFF printing.

PLA is a biodegradable material derived from renewable resources and possesses good mechanical properties, which makes it a promising and eco-friendly material for composite applications. In recent years, researchers mainly worked towards improving the mechanical properties of FFF printed parts. Some researchers investigated the nature of FFF and the effect of the process parameters [10,11] on the mechanical performances of FFF printed parts. Ali Chalgham et al. [12] investigated the influence of build orientation, layer thickness, printing temperature, printing speed, and heat treatment on the mechanical properties of the PLA samples. The effects of the layer thickness, part orientation, raster angle, raster width, and air gap process parameters on the tensile performance has also been investigated by Onwubolu [13]. From their results, tensile strength was highest when the layer thickness is lowest, and the part orientation was printed parallel to the direction of the applied tensile force. Furthermore, tensile strength increased at a higher raster angle with a low raster width and at a negative air gap, which is in similar agreement with Dawoud et al. [14]. Pang et al. [15] investigated the effect of the printing temperature of PLA on bonding quality and tensile strength of FFF printed parts. As the printing temperature increased, the tensile strength also increased, but poor part quality can be observed. This is due to high fluidity and low viscosity, which leads to poor dimensional accuracy. Thus, the selection of printing parameters is crucial according to the feedstock material. Moradi et al. [16] used statistical analysis and a response surface methodology (RSM) with an experimental approach in other to evaluate the optimum layer thickness, infill percentage, and number contour for Nylon FFF printed parts. However, with the optimum process parameters, FFF printed parts with neat polymer are still incomparable with synthetic material mechanical performances.

Therefore, some investigations studied the material aspects by adding the fibers or particles into the thermoplastic matrix to improve the strength performances. Gray Iv et al. [17] added thermotropic liquid crystal-line polymer fibrils into PP to prepare a composite filament for FFF. A capillary rheometer was used to simulate the FFF process, and subsequently, the tensile property of the extruded strands improved. Zhong et al. [18] studied the FFF process of short fiberglass-reinforced ABS. The additions of plasticizer and compatibilizer improved filament processibility. They also conducted experiments to investigate the processability of fiberglass-reinforced ABS matrix composites with three different fiber contents used as filaments in FFF. The results showed that fiberglass could significantly improve the tensile strength and surface rigidity of the ABS filament. Furthermore, Shofner et al. [19] investigated the effects of vapor-grown carbon fibers added into ABS as FFF filaments on the mechanical properties. An 39% average increase in tensile strength was observed at a 10 wt% nanofiber loading. Tekinalp et al. [6] reinforced ABS by adding short carbon fibers to investigate its processibility, microstructure, and mechanical performances. The tensile strength and modulus of 3D printed samples increased by 115% and 700%, respectively, and the fabricated samples’ carbon fiber orientation was up to 91.5% in the printing direction. PLA’s strength, rigidity, and toughness may be coincidentally improved by adding glass fibers [20]. Based on previous studies, adding nanomaterials such as carbon nanotubes, nanowires, and nanoparticles to thermoplastics via FFF seems to have the potential to improve the performances of the resulting printed parts [3,18].

The performance of glass fiber-reinforced thermoplastics depends not only on the properties of the matrix and fiber, but is also affected by the amount of glass fibers, orientation, aspect ratio of the fibers, distribution, and fiber-matrix adhesion [21,22]. Thus, fiber content is one of the most fundamental quantities controlling the properties of fiber-reinforced composites. Stiffness, strength, and other properties depend mostly on the fiber volume fraction and orientation. The relationship of the mechanical properties of the composites and the fiber volume fraction experimental results were compared with the mathematical model of the “Rule of Mixtures” that had been previously used to predict the mechanical properties of the composites [23]. The elastic modulus of the discontinuous and randomly oriented-fiber composite E_c_ was determined using the Equation (1) below [24]:

E_c_ = K(E_F_V_F_) + (E_M_V_M_) (1)

where K = reinforcement efficiency, E_F_ = elastic modulus of fiber, V_F_ = percentage volume of fiber, E_M_ = elastic modulus of matrix, and V_M_ = percentage volume of the matrix.

Table 1 depicts the short fiberglass with different fiber volume fractions from previous research. In order to investigate the volume fraction of fiberglass, thermogravimetric analysis (TGA) has been used. It is known that the ignition method is suitable and quicker to measure glass fiber content in the composites [25]. This is because glass fiber and thermoplastics significantly differ in melting points.

From previous studies of short fiber-reinforced polymer in FFF, three different methods can be used to produce 3D printed composites, and a detailed review was summarized in a recent work [9]. The most common method to print a 3D composite is by using a composite filament, where the fibers are embedded into thermoplastic to form composite filaments prior to printing, which is method 1 (M1). The second method is by mixing short fiber and thermoplastic inside a 3D printer extruder (M2). However, there is no study on embedding the short fiber on the printed part during the printing process, which is method 3 (M3). The mechanical performance of the short fiber-reinforced composite using M1 and M2 improved, but voids between the interlamellar bonds still exist [9]. Therefore, the short fiber-reinforced composite using M3 has been introduced in this work. Theoretically, the deposition of reinforcement, such as glass fiber powder, between the deposited filaments will reduce the voids which exist between the deposited filaments and act as a bridge to improve the layer-to-layer bond between the deposited filaments. A new method of embedding fibers on molten-deposited thermoplastic filament during the printing process was proposed recently [28]. The present work aims to investigate the tensile properties of the in situ-manufactured GFPLA composites, manufactured using the newly proposed method and compared with the non-reinforced counterpart, the neat PLA. The fracture surfaces of all samples are observed using a SEM. The porosities of the 3D printed samples are also measured with an image processing software. The correlation between the results are also identified. 

## 2. Materials and Methods

### 2.1. Raw Materials

The PLA polymer used in this work is from Forcemaker3D (Nazca Scientific Sdn. Bhd, Cheras, Malaysia) and the milled E-glass fiber type was supplied by Shenzhen Feige Composite Fiber Co., Ltd., based in Shenzhen, China. PLA is favored for its biodegradability, absence of unpleasant odors when heated, and overall environmental compatibility in all aspects of its life cycle [23]. The milled E-glass fiber was made from E-glass strands chopped via a ball mill, followed by wet grinding, drying, and screening. These procedures are commonly used to reinforce unsaturated, epoxy, and phenolic resins. It can improve foaming power for coating, which has fast impregnation, good dispersion, and less wool-making material which has excellent mechanical properties. This product meets the Restriction of Hazardous Substances (ROHS) standard. Table 2 below shows the specifications of the glass fiber used in this research work.

Glass fiber is a type of synthetic material that significantly improves the strength of printed parts [14]. Short glass fiber-reinforced polymer (SGFRP) material is widely used as a structural material in many engineering applications, as it offers several advantages such as higher strength and the ability to recycle. Therefore, short glass fiber-reinforced (SGF) plastics are of great commercial and scientific interest. Based on the glass fiber specification provided by the supplier, the glass fiber was already treated with silane as a coupling agent. Milled glass fiber without a coupling agent improves the elastic modulus but reduces the tensile and impact strength properties below the polymer matrix’s value [29].

### 2.2. Composite Production

In this work, the 3D printer consists of two extruders, where one extruder deposits the neat PLA, and the other extruder, known as a fiber doser, deposits the milled glass fiber [28]. To fabricate the GFPLA composites, a novel fiber doser was designed and fabricated to deposit the glass fiber powder during printing [28]. The deposition rate of the glass fiber doser can be adjusted by controlling the motor speed of the fiber doser. The fiber doser was installed beside the printer nozzle, where the PLA thermoplastic material is extruded through a hot nozzle from the primary extruder. The tensile specimens were printed in dog-bone shapes according to the ASTM D638-14 [30] Type 1. Section 6.1.3 of the ASTM D638-14 recommends that the reinforced composites, including highly orthotropic laminates, should conform to the dimensions of the Type 1 specimen. Type 1 was also chosen because it has a larger cross-section area, allowing more reinforcing material to be deposited. 3D printing was performed on a Forcemaker3D printer with a nozzle diameter of 0.4 mm, using PLA filament with a diameter of 1.75 mm. Printing velocity was set at 60 mm/min, with a layer thickness of 0.2 mm. The infill pattern deposition directions for different layers were 45° alternately. This infill pattern was selected to investigate the potential of the enhancement of layer-to-layer bonding via glass fibers. The infill density was set at 100%. A previous study by Nashruffi et al. [31] on the effect of printing orientation to the tensile properties showed that an on-edge build orientation had the greatest tensile strength, followed by a flat and upright orientation. However, a flat orientation has been selected in this work due to the larger reinforced area and anisotropic behavior of FFF printed parts. Five specimens were fabricated for each composite. Table 3 depicts the standard parameters in this study.

**Figure 1:**
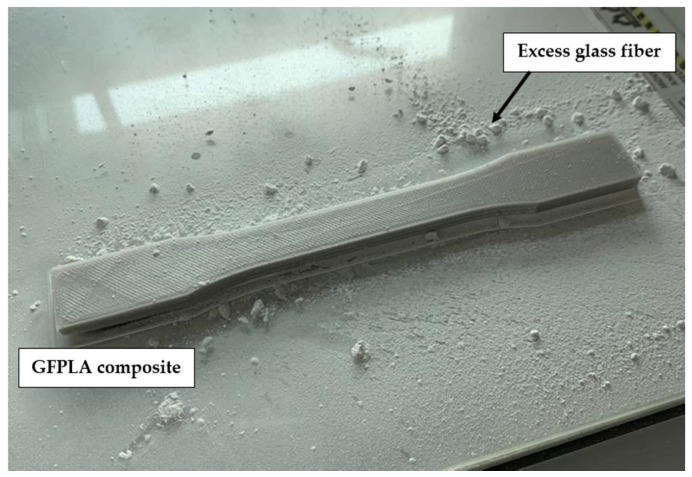
Printed dog-bone shape of the GFPLA composite.

**Figure 2:**
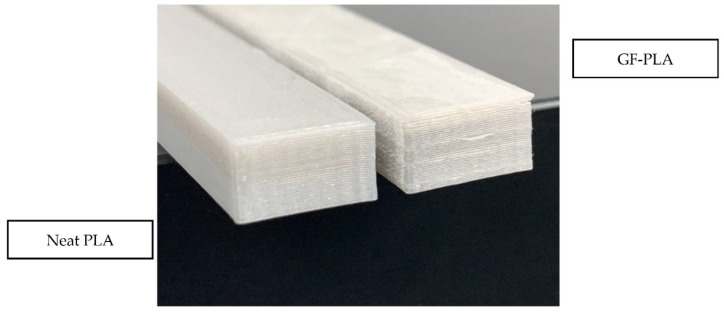
Printed samples for the neat PLA and GFPLA composite [28].

One neat PLA and 3 GFPLA composites with different glass fiber contents were fabricated for this tensile test: Neat PLA, GFPLA-1, GFPLA-2.4, and GFPLA-5. The GFPLA composites were differentiated via the fiber doser motor speed. Glass fiber content in each composite was then identified using the TGA [28]. The glass fiber content for all samples is presented in Table 4. After multiple investigations, the extrusion temperature was set at 210 °C to prevent the specimens from warping and distorting during printing. Several fabrication attempts have been made to fabricate GFPLA in order to obtain suitable nozzle temperature. Overheating causes the material or extrudate to degrade, making it unable to retain its shape upon deposition and resulting in deformation and distortions to the dimensional accuracy. On the other hand, if the extrusion temperature is low, the material does not have enough time to fully melt, which results in the clogging of the nozzle and delamination between the deposited layers. The addition of glass fibers also affects the selection of optimum nozzle temperature, which causes decoupling between the PLA layers if PLA cools too quickly. Figure 1 shows a printed part of the GFPLA composite, while in Figure 2, it illustrates the neat PLA and GFPLA composite specimens for the tensile test.

### 2.3. Tensile Test

The tensile test was conducted using a universal test machine (Galdabini Tester Quasar 10, Cardano al Campo, Italy) with a 10 kN force transducer capacity. The tensile test specimens were held by two grips (one fixed grip and one movable grip). The tensile test was performed with five repetitions for each composite. An axial extensometer (Reliant Technology, Colorado Springs, CO, USA) was used to measure strain, and the testing speed was set to 5 mm/min. The tensile modulus, E_t_, was calculated from the slope of the stress–strain curve. The relationship between stress and strain was generated via a computer using data acquisition software (Graphwork 5.0, Galdabini, Cardano al Campo, Italy). Five tensile tests were conducted for each condition, and the average value was calculated for tensile strength, tensile modulus, and elongation at break. The average, standard deviation and standard error were calculated using the method by Shamsuri et al. [32]. The average (or the mean) value is the sum of all data collected and divided by the number of data (*n*) collected. The standard deviation is a measure of the amount of variation in the dataset, and the relative standard deviation shows the deviation of a set of numbers disseminated around the mean, as shown below:

(2) $$\mathrm{Average},\;\bar{x}=\frac{\left(x_{1}+x_{2}+\cdots +x_{n}\right)}{n}$$

(3) $$\mathrm{The}\;\mathrm{standard}\;\mathrm{deviation}\;S=\sqrt{\frac{\sum_{i=1}^{n}{\left(x_{i}-\bar{x}\right)}^{2}}{n-1}}$$

(4) $$\mathrm{The}\;\mathrm{relative}\;\mathrm{standard}\;\mathrm{deviation},\;\mathrm{RSD}=\frac{S}{\bar{x}}$$

After the tensile test was completed, the fractured surfaces of the specimens were observed under SEM with different magnifications to investigate the fracture mechanism and effects of fiberglass on the interlamellar bonds. 

### 2.4. Surface Morphology

The fractured surfaces of the tensile specimens were observed using a scanning electron microscope (LEO 1455VP SEM) with different magnifications to investigate the fracture mechanism and effects of fiberglass on the interlamellar bonds. Layer height, L_h_, and line width, L_w_, were also calculated and compared with the intended layer thickness to investigate the presence of glass fibers within the extruded filament. ImageJ version 1.53t software was used to measure L_w_ and L_h_ of the individual filaments. After that, FESEM (JEOL JSM-7600F, Japan) was used to analyze the porosity of the neat PLA and GFPLA composites. All specimens were coated with gold prior to imaging to provide conductive surfaces. The area of porosity was calculated using the threshold method by the ImageJ version 1.53t. For ImageJ analysis, the threshold method was used to identify the porosity. The dark region was identified as porosity and the summation of the dark region areas was measured.

## 3. Results 

### 3.1. SEM Images of Glass Fiber

**Figure 3:**
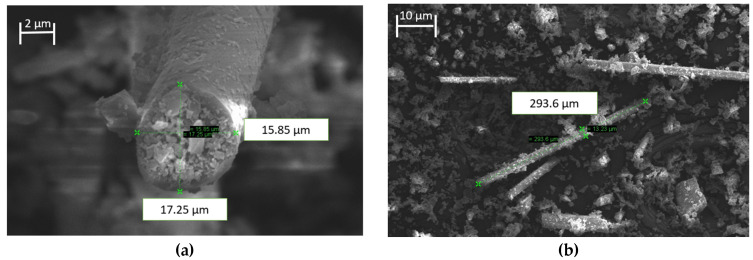
SEM image of the glass fiber: (**a**) fiber diameter, and (**b**) fiber length.

The SEM images of the milled glass fiber with an estimated average fiber diameter of 13 µm and fiber length of 160 µm used in this research work are illustrated in Figure 3. The SEM images were obtained using a LEO-1455VP electronic microscope, operating with a 20 kV electronic beam. Carbon tape was used to hold the milled glass fiber before it was observed under SEM. These images confirmed that the milled glass fiber received was correct with the specification given by supplier.

### 3.2. Tensile Properties

**Figure 4:**
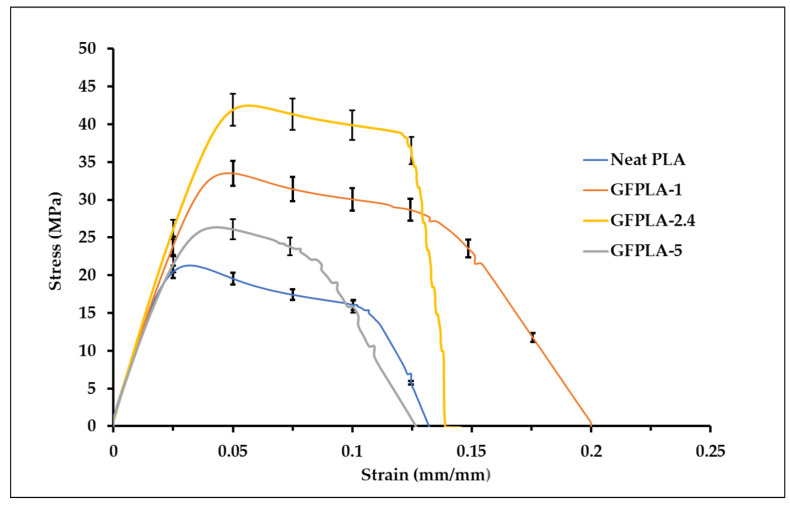
Stress–strain curve of the neat PLA, GFPLA-1, GFPLA-2.4, and GFPLA-5. Error bars indicate standard errors in five tests.

The typical tensile stress–strain curves for the neat PLA, GFPLA-1, GFPLA-2.4, and GFPLA-5 are illustrated in Figure 4. Each curve was selected from the results of the five repetitions, depending on the maximum number of values closest to the mean value of each maximum tensile stress. The error bars represent one standard deviation at the selected strain values. Due to the unsynchronized stress and strain data for each specimen, the average and standard deviation values were calculated according to each selected strain (mm/mm). Based on the following tensile stress–strain curves, it can be seen that tensile stress increased as the fiberglass content increased from 1.05 wt% in GFPLA-1 to 2.39 wt% in GFPLA-2.4. The tensile stress drastically dropped at GFPLA-5 (4.98 wt%) but it is still higher than the neat PLA. When the glass fiber content was at 1.05 wt%, it showed ductility characteristics. However, as the glass fiber content increased to 2.39 wt% and 4.98 wt%, the composites became more brittle. 

**Figure 5:**
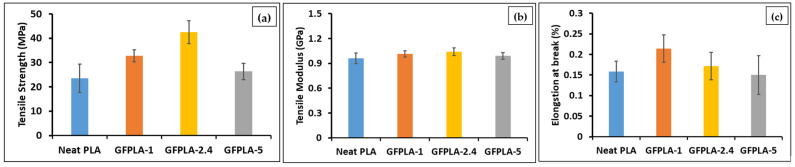
(**a**) Tensile strength and (**b**) tensile modulus (**c**) elongation at break. Error bars indicate standard errors in five tests.

Figure 5 shows the tensile performances of the neat PLA and GFPLA composites. Low relative standard deviation values were obtained. Relative standard deviations values were below 5%, which means the results were nearer to the average values. In general, the tensile strength of GFPLA increased with the inclusion of glass fibers. GFPLA-2.4 resulted in the highest tensile strength with an 80.7% improvement compared to the neat PLA, whereas composites GFPLA-1 and GFPLA-5 showed a 39.2% and a 12.08% improvement, respectively. The GFPLA-1 and GFPLA-2.4 curves showed ductile behavior as both composites failed at the higher strains. However, composite GFPLA-5, with the highest glass fiber content, showed a brittle behavior. Based on the trend, GFPLA-2.4 provides the optimum glass fiber content based on tensile strength. The tensile modulus for all samples is presented in Figure 5b, and the GFPLA samples exhibited slightly higher tensile modules, E_t_, than the neat PLA samples. This increased stiffness suggested that the fiberglass-reinforced samples showed greater resistance to plastic deformation due to the effective load transfer to the fiberglass. The elongation at break improved by 35.3% and 8.2% for GFPLA-1 and GFPLA-2.4, respectively, when compared to the neat PLA; however, it reduced by 5.2% for GFPLA-5 as the composite became more brittle.

**Figure 6:**
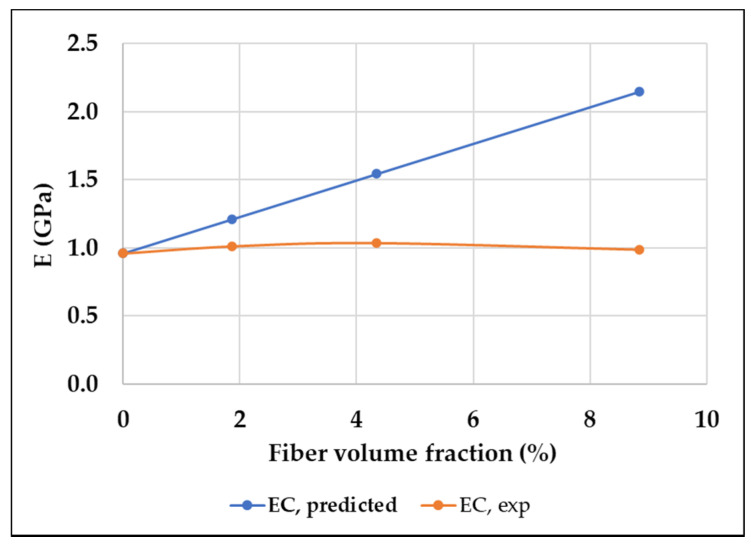
E_c_ predicted and experimental versus fiber volume fraction.

The elastic modulus of the composites E_C_ was calculated according to the rule of mixtures (Equation (1)) and compared with the experimental values in Table 5 and Figure 6. For fibers randomly and uniformly distributed within three dimensions in space, the reinforcement efficiency K is 0.2 [24], the elastic modulus of the glass fiber = 72 GPa [33], and the elastic modulus of the 3D printed PLA = 0.96 GPa. 

Additionally, Figure 6 also shows that the E_c_ (experimental) increased with the increased glass fiber content but dropped at the maximum glass fiber content, whereas, for E_c_ calculated via the mathematical model, the value keeps increasing as the fiber content increases. The decrease in tensile strength of the composite with the highest glass fiber content is caused by the weaker interlayer bonds and will be discussed in Section 4.2. 

### 3.3. Observation of Fracture Surfaces 

**Figure 7:**
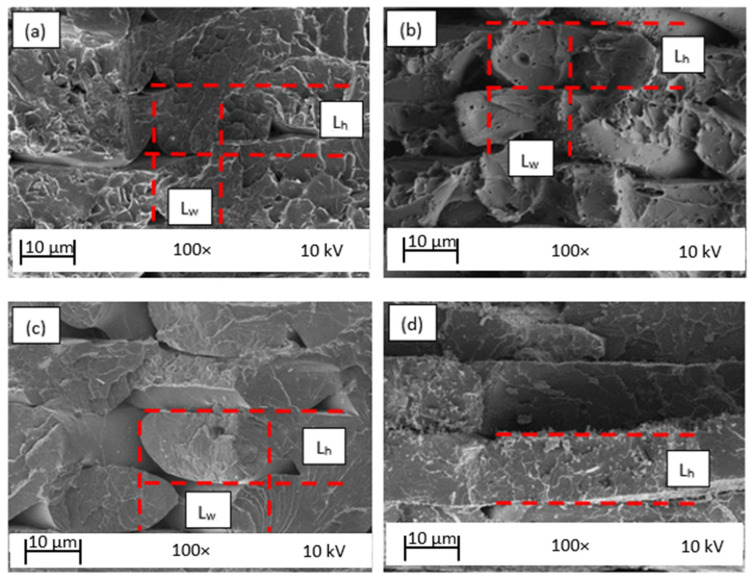
(**a**) Neat PLA, (**b**) GFPLA-1, (**c**) GFPLA-2.4, and (**d**) GFPLA-5 obtained via SEM under 100× magnification, operating with a 10 kV; L_h_ is the layer heights of the deposited material (the road) and L_w_ is the width of the road.

**Figure 8:**
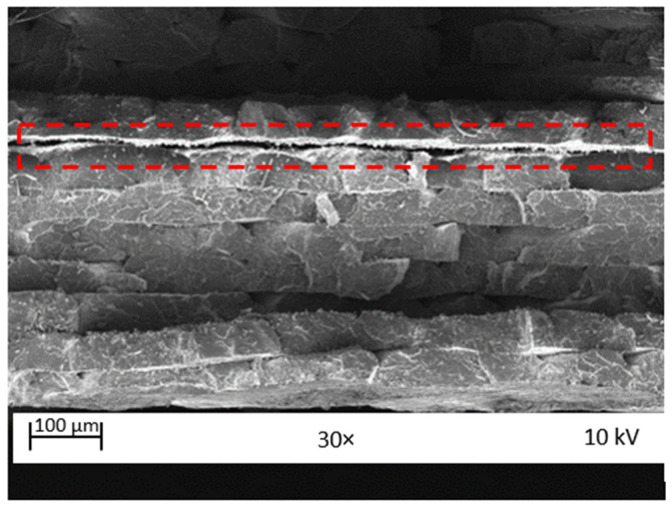
SEM image of the GFPLA-5 fracture surface.

The fractured surfaces of the neat PLA and GFPLA composite specimens with different glass fiber contents after tensile testing were observed using SEM to (i) investigate the fracture behavior and (ii) explore the specimen porosity and interfacial adhesion between PLA-PLA and GF-PLA. The SEM examination of the cross-sectional tensile-fractured surfaces of the 3D printed GFPLA specimens is depicted in Figure 7. This study was performed to gather data on the effects of different fiberglass contents on the morphology of the deposited strands. Four specimens were observed: neat PLA, GFPLA-1, GFPLA-2.4, and GFPLA-5. Figure 8 shows delamination occurred due to a high concentration of glass fibers. 

**Figure 9:**
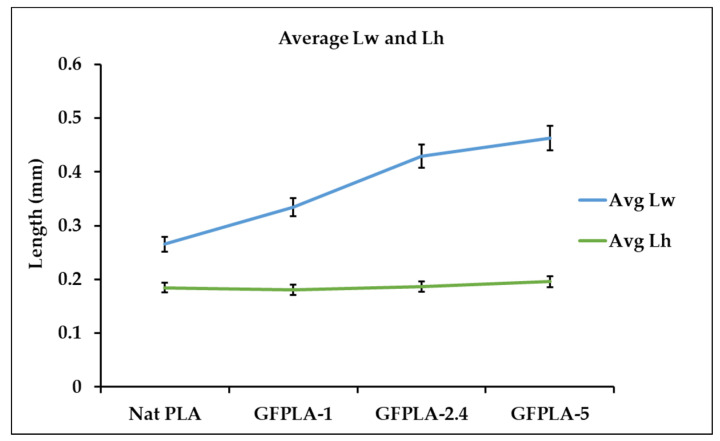
Average line width (L_w_) and layer height (L_h_) of the roads from different composites. Error bars indicate standard errors in six measurements.

From the SEM micrographs in Figure 7, it can be observed that the porosity between the layers of the GFPLA composites reduced with the increased glass fiber content. The line width L_w_ and layer height L_h_ of the roads of all composites were measured using ImageJ, and then the average of six measurements is plotted in Figure 9. As shown in Figure 9, the average L_w_ of the deposited material increased as glass fiber content increased, while L_h_ remained at ~0.2 mm. This indicates that the presence of glass fiber improved the intralayer bonds but reduced the interlayer bonds. As shown in Figure 7, each layer at GFPLA-5 became even, which made it difficult to identify the value of L_w_. The SEM image of GFPLA-5 (Figure 7d) shows that the deposited filament completely bonds within the same layer. 

**Figure 10:**
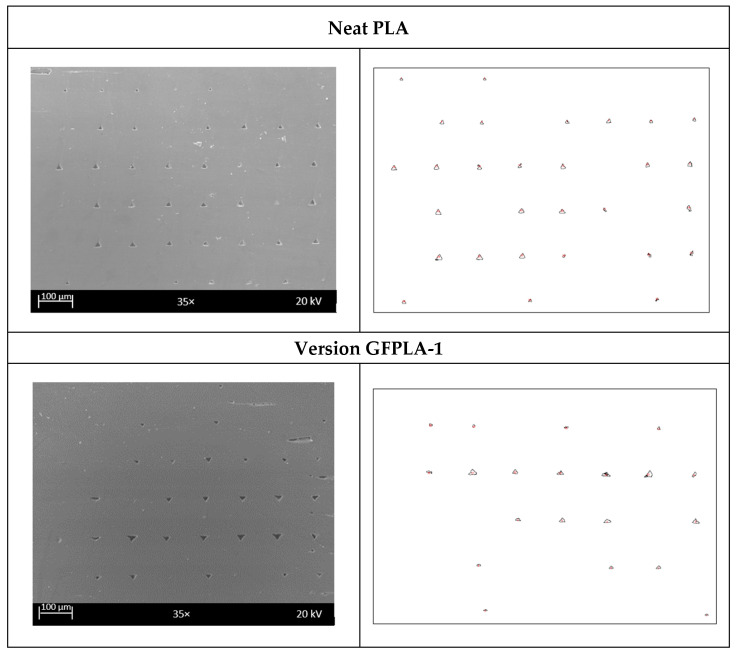

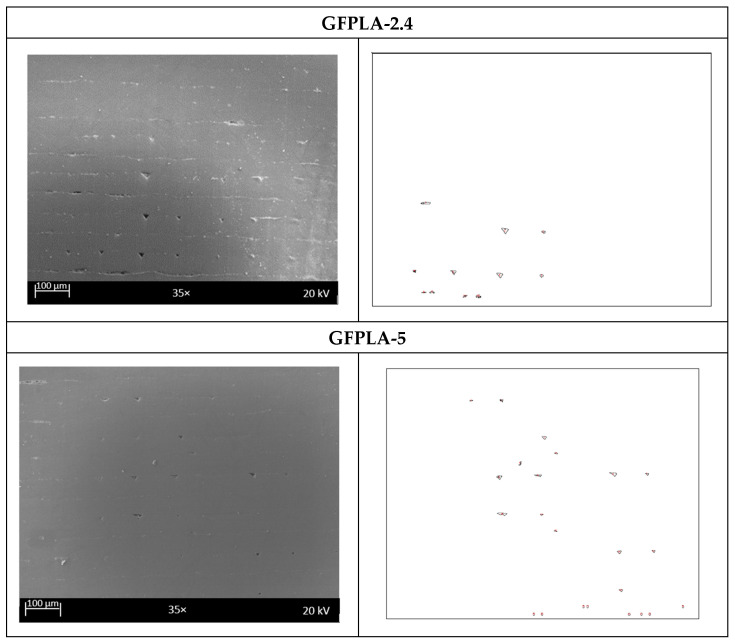
Porosity identified by using Image J version 1.53t.

Although the difference in layer height for the neat PLA and the composites is not significant in value, the SEM images from Figure 7 show that the shapes of the deposited filament were deformed from oval to rounded rectangular, and the porosity was reduced with the deposited filaments. Figure 10 shows the SEM images for each specimen and the porosity identified by ImageJ. All images were taken under 35× magnification. By adjusting the threshold, ImageJ automatically identified the darker regions as porosity. The number of porosity and the porosity area were calculated. The left side of Figure 10 shows the original SEM images for each composite, while the right side shows porosity identified using the ImageJ version 1.53t.

**Figure 11:**
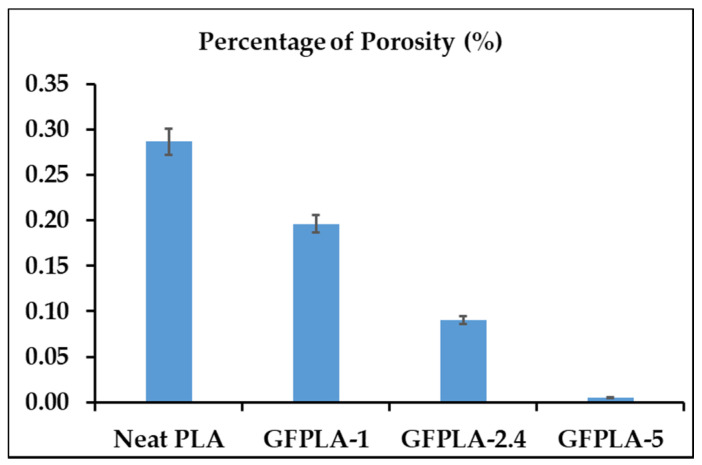
The percentage of porosity in respect to glass fiber content. Error bars indicate standard errors in three repetitions using the same SEM image for each composite, but different setting size (pixel^2^) in ImageJ.

**Figure 12:**
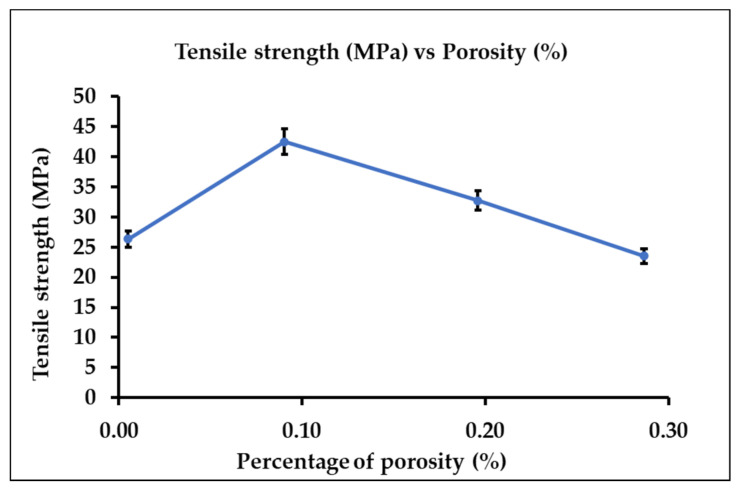
Tensile strength in respect to percentage of porosity. Error bars indicate standard errors in three repetitions.

From the ImageJ analysis, the total porosity area was calculated for each composite. Based on Figure 11, the trend shows that the porosity area reduced as the glass fiber content increased. This concludes that the fiberglass concentration will affect the area of porosity. Furthermore, Figure 12 depicts tensile strength trends as the porosity of the GFPLA composite increases. When the percentage of porosity reduced from 0.29% to 0.09%, the tensile strength increased to a maximum of 42.48 MPa. However, further reduction in porosity shows a decreasing trend of the tensile performance of the GFPLA composite. 

## 4. Discussion

### 4.1. Tensile Properties

The stress strain curves presented in Figure 4 indicate that the addition of glass fibers to PLA alters the tensile behavior of PLA. It seems that the interlamellar bonds improved as the glass fiber content increased up to 2.39 wt%. However, at 4.98 wt% glass fiber content, the tensile performance deteriorated due to the high amounts of glass fiber which reduces heat travel within the deposited PLA to form the interlamellar bonds. GFPLA-1 and GFPLA-2.4 curves showed ductile behavior as both composites failed at the higher strains. However, composite GFPLA-5 with the highest glass fiber content showed a brittle behavior. Based on the trend, GFPLA-2.4 provides the optimum glass fiber content based on tensile strength. A similar trend was reported by Rinaldi et al. [34], where 3 wt% of CNT in PEEK is the optimum reinforcement content for tensile strength.

In general, the E obtained from the experiment is lower than the calculated E, and a similar trend was reported by [34,35,36,37]. The difference between the theoretical E_c_ and experimental E_c_ is mainly caused by the fabrication method used in the current work, where a 3D printer prints the samples with porosity, but the rule of mixtures do not consider porosity in the composite. Moreover, Equation (1) (rule of mixtures) is based on a pure micromechanic approach [34] where the assumptions are no longer valid in the current study. As highlighted by [34], three assumptions of the rule of mixtures are not valid in the FFF 3D printed composite:

(1) Strong interface: the rule of mixtures assumes that there is a strong interface between the reinforcement (glass fiber) and the matrix material (PLA, in this case). In reality, the interface strength might not be ideal, which can influence the mechanical properties of the composite.
(2) Isostrain conditions: the rule of mixtures assumes that both the matrix and the reinforcement are under isostrain conditions, meaning they deform together without any relative movement. In practical situations, this assumption might not hold true and could affect the overall mechanical behavior of the composite.
(3) Homogenous and random fiber distribution: the rule of mixtures assumes that the fibers are homogenously distributed and randomly oriented within the matrix. In real-world scenarios, achieving perfect homogeneity and random orientation can be challenging during the 3D printing process, leading to deviations from the idealized model.

Furthermore, the rule of mixtures also does not consider the nucleating effect of glass fibers. Glass fiber has a remarkable nucleating effect that can significantly improve the crystallization of PLA [20]. Compared to the neat PLA, the GFPLA composites have a lower crystallization temperature, which means that they require less energy to reach the crystallization stage. This is due to the nucleating effect of glass fibers, which accelerates the crystallization process and shortens the time needed to complete it. The faster crystallization process resulting from the inclusion of glass fibers is highly advantageous as it contributes to improving the mechanical properties. Crystalline PLA phases are known to be stronger and stiffer than amorphous PLA phases. Therefore, the nucleation and growth of crystalline structures in the GFPLA composites lead to increased strength [20], as observed in Figure 5a, where an addition of 1.02% of glass fibers improved the tensile strength to 39.2%. In addition, increasing the glass fiber content in the PLA composites can further promote crystallization and reduce the time required for the material to crystallize fully. Therefore, increasing the glass fiber to 2.4% further improved the tensile strength and achieved an 80.7% improvement. However, a further increase in glass fiber prevented the α-spherulites of PLA from expanding in all directions, thus resulting in a decrease in crystallinity, similar to previous findings [38].

### 4.2. Observation of Fracture Surfaces

From the images of the neat PLA (Figure 7a), GFPLA-1 (Figure 7b), and GFPLA-2.4 (Figure 7c), the formation of voids can obviously be seen between the deposited filaments. These voids were formed due to the natural layer-wise FFF printing process [29,39,40,41]. The voids occurred as a result of the rapid cooling rate, which caused a incomplete neck growth between the deposited filaments [9]. However, Figure 7d shows a complete neck growth within the intralayer bond, which reduced the size of the voids. Furthermore, GFPLA-1 (Figure 7b) and GFPLA-2.4 (Figure 7c) composites showed greater plastic deformation. Contrary to this, the GFPLA-5’s results (Figure 7d) showed a brittle behavior where the fracture surface was contained on the same plane, which is consistent with the findings of Caminero et al. [42]. The high glass fiber may deteriorate the interlayer bond, which causes a drop in the tensile strength of the GFPLA-5 composite.

### 4.3. The Functions of Glass Fiber

As mentioned in Section 4.1, glass fiber has a remarkable nucleating effect that can significantly improve the crystallization of PLA. The presence of glass fibers in the PLA composite induces a nucleating effect and increases the crystallization process. Crystalline PLA phases are recognized for exhibiting superior strength and rigidity when contrasted with amorphous PLA phases. This in turn leads to an enhancement in strength through the initiation and growth of crystalline structures within the GFPLA composites [20].

Furthermore, milled E-glass fiber has a lower thermal conductivity than PLA, which is 0.03 Wm^−1^K^−1^, while PLA is 0.185 Wm^−1^K^−1^ [43]. The heat transfer occurs at a lower rate in materials of low thermal conductivity than in materials of higher thermal conductivity. When a large amount of glass fiber was deposited on the surface of the hot extruded PLA-deposited material, the surface temperature of the PLA road was reduced because of the thermal conduction from the hot PLA to the glass fiber. 

**Figure 13:**
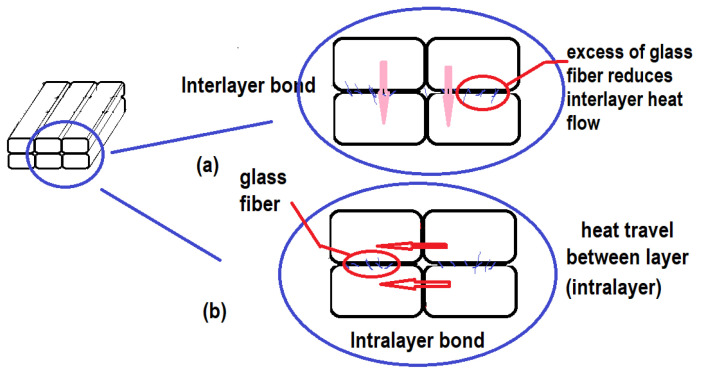
(**a**) Excess of glass fiber reduced neck growth, and (**b**) Heat flow between the layer (intralayer).

Due to the sequence of printing, the previously deposited layer (bottom PLA layer) has a lower temperature compared with the newly deposited layer. Furthermore, a large amount of glass fiber reduces the heat flow from the upper PLA layer to the previously deposited layer (bottom PLA layer). As mentioned previously, milled E-glass fiber has a lower thermal conductivity than PLA. A larger amount of glass fibers lowers the surface temperature of PLA and reduces the interlayer heat transfer required to create a strong bond between the newly deposited layer with the previously deposited layer/bottom PLA layer (interlayer bond). Hence, delamination between the layers can be observed from the fractured specimens in GFPLA-5. High glass fiber powder creates a border which prevents heat transfer required for neck growth between the deposited filaments, weakening the interlayer bonds, as shown in Figure 13a. Meanwhile, milled E-glass fiber was not present between the adjacent filaments, and as such, heat was transferred between the adjacent filaments within the same layer, as shown in Figure 13b, which increases the intralayer bonds. 

As shown in Figure 11, the area of porosity decreased when the glass fiber content increased. As mentioned earlier, the deposited filament was deformed from an oval to a rounded rectangular when the glass fiber content increased. This indicated that the addition of glass fibers altered the temperature distribution of the subsystem and further affected the rheology of the deposited filament [44,45]. As mentioned previously, the addition of glass fibers changed the temperature distribution of the sub-system and then affected the rheology of the deposited filament. At the same time, the excess of glass fiber reduced the surface temperature of PLA and slowed down the interlayer heat transfer required to create a strong bond between the two deposited layers (interlayer bond).

## 5. Conclusions

In summary, this work analyzes the effect of in situ glass fiber reinforcement using a modified Fused Filament Fabrication 3D printer with a fiber doser on the printed part’s layer-to-layer bonding and tensile performance.

(1) The tensile strength increases with an increase in the glass fiber reinforcement up to a limit of 2.39 wt%. Afterwards, the tensile strength reduces with the increased fiber content, where the presenting values are almost similar to those of pure thermoplastic.
(2) Effects of glass fiber reinforcement on the elastic modulus presented a behavior similar to tensile strength. However, the smallest elastic modulus value was found for the neat PLA.
(3) From the morphology analysis, the presence of in situ glass fibers improved the intralayer bonds but reduced the interlayer bonds of the deposited filament. This is because the inclusion of glass fiber reduced heat transfer between the PLA-deposited layers, agitating neck growth between the PLA layers. The shapes of the deposited filament were deformed from oval to rounded rectangular, and the porosities in the printed samples were reduced with the increase in glass fiber in the PLA composite. This indicates that the inclusion of glass fiber affected the temperature distribution of the subsystem and then the rheology of the deposited PLA.
(4) The new printing method using the fiber doser improved the tensile performance by reducing the void between the deposited PLA layers; however, excessive fiber content weakened the interlayer bond and deteriorated the tensile performance.

## Figures and Tables

**Table 1:** Properties of short fiberglass by the pre-mixed filament (M1).

Polymer	Fiber Content (%)	Strength (MPa)	Modulus (GPa)	Ref.
PP	30	30–35	-	[26]
PP	-	45–50	5–8.9	[21]
PP	30	32	0.95–1.5	[7]
ABS	10, 20, 30	43.4–93	2.24–8.41	[27]
ABS	15, 20, 25, 30	58.6	-	[18]

**Table 2:** Glass fiber specifications used in this research work.

Specifications	Average Value
Model	MEF-13-100
Color	White
Glass type	E-Glass
Mesh	100
Fiber diameter	13 µm
Fiber length	160 µm
Aspect ratio	12:1
Bulk density	0.67 g/cc
Moisture content	<1.5%
Loss of ignition	<1%
Alkali content/R2O (%)	<0.80
Sizing	Silane
Contamination	Free from dirt, lumps, unmilled fiber

**Table 3:** Printing parameters.

Parameter	Standard Value
Nozzle temperature (°C)	210
Heating bed temperature (°C)	70
Number of shells	3
Infill pattern	Rectilinear
Infill density (%)	100
Raster angle (°)	[+45/−45]
Layer thickness (mm)	0.2
Printing speed (mm/min)	60
Build orientation	Flat

**Table 4:** Content of glass fiber in the specimen.

Name	Contents of Glass Fiber (Mass Fraction, %)
PLA	0
GFPLA-1	1.02
GFPLA-2.4	2.39
GFPLA-5	4.98

**Table 5:** Analytical result of E_C._

Sample	GF Content (Mass Fraction, wt%)	GF Content (Volume Fraction, %)	E_c_ (Predicted)	E_c_ (Experimental)
PLA	0	0	0.96	0.96
GFPLA-1	1.02	1.87	1.2113	1.0133
GFPLA-2.4	2.39	4.34	1.5433	1.0387
GFPLA-5	4.98	8.84	2.1481	0.9892

## Data Availability

The data presented in this study are available on request from the corresponding authors.
